# Understanding the Role of Metakaolin towards Mitigating the Shrinkage Behavior of Alkali-Activated Slag

**DOI:** 10.3390/ma14226962

**Published:** 2021-11-17

**Authors:** Bo Fu, Zhenyun Cheng, Jingyun Han, Ning Li

**Affiliations:** 1 School of Civil Engineering, North Minzu University, Yinchuan 750021, China; 2015006@nmu.edu.cn (B.F.); 2016035@nmu.edu.cn (Z.C.); 2014046@nmu.edu.cn (J.H.); 2 National Energy Group, Coal Chemical Industry Technology Research Institute, Ningxia Coal Industry Co. Ltd., Yinchuan 720021, China; 3 College of Civil Engineering, Hunan University, Changsha 410082, China; 4 School of Engineering, University of Glasgow, Glasgow G12 8LT, UK

**Keywords:** alkali-activated slag, metakaolin, compressive strength, autogenous shrinkage, drying shrinkage, pore structure

## Abstract

This research investigates the mechanism of metakaolin for mitigating the autogenous and drying shrinkages of alkali-activated slag with regard to the activator parameters, including concentration and modulus. The results indicate that the incorporation of metakaolin can decrease the initial viscosity and setting time. Increasing activator concentration can promote the reaction process and shorten the setting time. An increase in the metakaolin content induces a decrease in compressive strength due to reduced formation of reaction products. However, increasing activator dosage and modulus can improve the compressive strength of alkali-activated slag containing 30% metakaolin. The inclusion of metakaolin can mitigate the autogenous and drying shrinkage of alkali-activated slag by coarsening the pore structure. On the other hand, increases in activator concentration and modulus result in an increase in magnitude of the autogenous and drying shrinkage of alkali-activated slag containing metakaolin. The influence of the activator modulus on the shrinkage behavior of alkali-activated slag-metakaolin binary system should be further investigated.

## 1. Introduction

Nowadays, alkali-activated slag is regarded as one of the most promising alternative low-carbon binders to Portland cement [1,2,3]. However, there are several disadvantages of alkali-activated slag, such as inferior workability [4], quick setting [5], and high shrinkage [6,7], although alkali-activated slag possesses high compressive strength [4], superior durability [8,9], and good fire resistance [10,11]. However, more intensive shrinkage behavior of alkali-activated slag than that of Portland cement, which induces a potential risk of cracking, limits the large-scale application of alkali-activated slag [12,13,14,15]. Generally, alkali-activated slag has about a 10-time and 6-time higher autogenous and drying shrinkage than Portland cement [16]. Li et al. [17] reported that alkali-activated samples cured in seal condition exhibited a 10-fold greater shrinkage than Portland cement-based samples.

There are four methods to achieve the mitigation of the shrinkage behavior of alkali-activated slag, which include internal curing [18,19], using shrinkage reducing admixtures or expansive agents [20], elevating curing temperature [21], and incorporating other silicon aluminum-base binders, such as fly ash [22], metakaolin [6], and waste glasses [23], respectively. Furthermore, in those strategies, the inclusion of other precursors present a better practicability from the perspective of cost consideration. Extensive research has reported that partial replacement of slag with fly ash could considerably reduce the shrinkage of alkali-activated slag by the filler effect of fly ash [22]. However, it is noted that the incorporation of fly ash would result in the degradation of the mechanical performance of alkali-activated slag. Chi et al. [24] reported that the compressive strength of alkali-activated slag sample with 50% fly ash cured for 28 d was almost 75% lower than that of pure alkali-activated slag samples. However, metakaolin has higher reactivity than fly ash [25]. Thus, it was considered that the incorporation of metakaolin would have a better mitigation effect on the shrinkage of alkali-activated slag [6]. Li et al. [6] reported that compared with pure alkali-activated slag, the autogenous shrinkage in an alkali-activated slag-metakaolin system was reduced and the mechanical performance did not significantly degrade, and considered that the inclusion of metakaolin could effectively inhibit the pore refinement, thus reducing the pore pressure. Although previous studies have confirmed the mitigation effect of metakaolin on autogenous shrinkage of alkali-activated slag, it is necessary to point out that the autogenous shrinkage of AAS is not only impacted by dosages of other precursors, but also by activator parameters, such as SiO_2_/Na_2_O ratios and Na_2_O contents and curing conditions [21,22,26]. However, to the authors’ best knowledge, limited studies involved with those aspects have been conducted [6,7].

Therefore, the current paper aims to systemically investigate the shrinkage behavior of alkali-activated slag with different metakaolin dosages, activator modulus, and Na_2_O contents. For this purpose, the viscosity, initial setting time, development of the compressive strength, autogenous and drying shrinkage, and the microstructure of alkali-activated slag pastes and mortars are studied. Additionally, the relationship between drying shrinkage characteristic and pore structure of alkali-activated slag-metakaolin system was investigated. The findings in this paper contribute to understanding of the roles of metakaolin in mitigating the autogenous and drying shrinkage of alkali-activated slag.

## 2. Experimental Programs

### 2.1. Raw Materials

**Figure 1:**
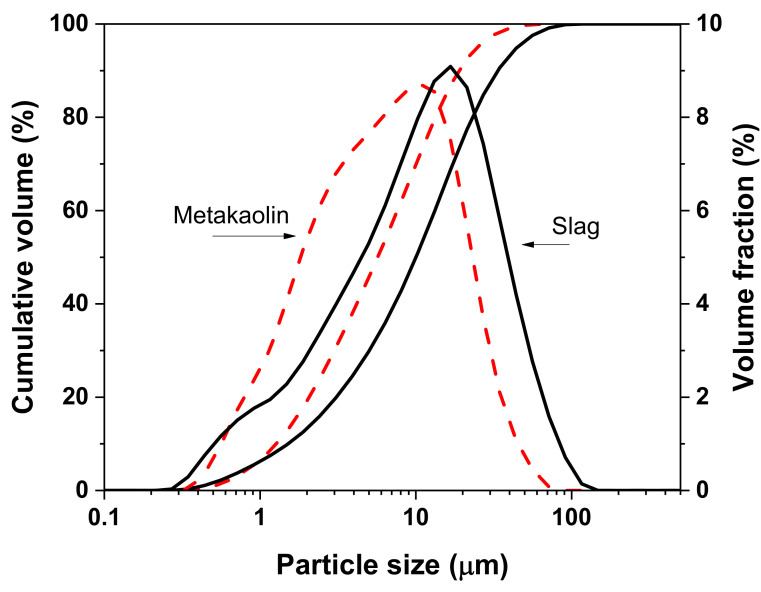
Particle size distributions of slag and metakaolin.

**Figure 2:**
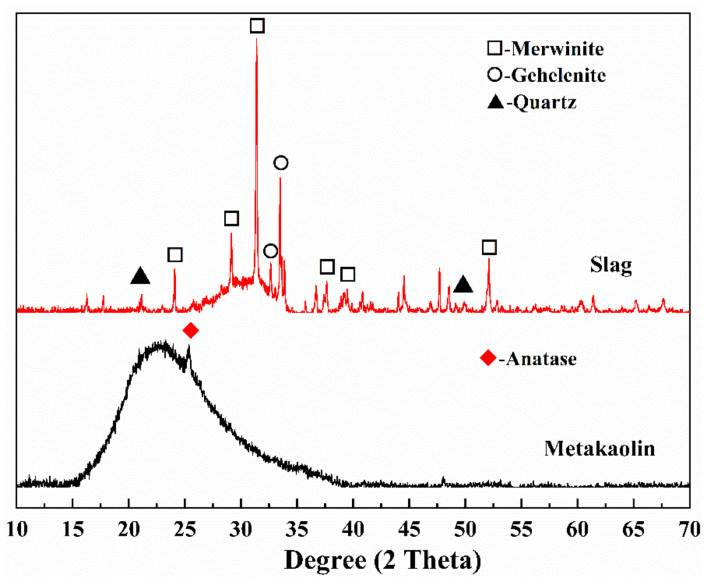
XRD patterns of slag and metakaolin.

Slag used in this study is ground granulated blast-furnace slag (GGBFS), provided from Ningxia Steel Group (NXSG), Zhongwei, China. Its specific surface area and density are 450 m^2^/kg and 2.70 g/cm^3^, respectively. Metakaolin supplied by Inner Mongolia Super New Material Co., Ltd., Huhhot, China was used. Its specific surface area was 491 m^2^/kg. The chemical compositions determined by X-ray fluorescence (XRF), particle size distribution determined by laser particle size analyzer, and phase compositions determined by X-ray diffraction are shown in Table 1 and Figure 1 and Figure 2, respectively. As depicted in Figure 2, there is an amorphous phase at 25–35° and the main crystalline phases of slag are merwinite (Ca_3_MgSi_2_O_8_, ICSD #26002), gehlenite (Ca_2_Al_2_SiO_7_, ICSD #158171), and quartz (SiO_2_, ICSD #174). In addition, there is less crystalline phase (Anatase, ICSD #1272) in metakaolin and an amorphous phase at 15–35° attributed to amorphous kaolinite [27], indicating the high reactivity of metakaolin.

Waterglass solution supplied by Lvseng Chemical Industrial Co., Ltd., Linfen, China containing 29.94 wt.% SiO_2_, 8.86 wt.% Na_2_O and 61.9 wt.% H_2_O was used in the current study. An analytically pure sodium hydroxide powder was also used. The standard sand in accordance with ISO679:1989 for preparing mortar samples was purchased from Xiamen ISO Standard Sand Co., Ltd. (Fujian, China). De-ionized water was used for the preparation of all samples in this study.

### 2.2. Mixture Proportions and Specimen Preparation

The activators used in this study were prepared by blending sodium hydroxide, waterglass solution, and de-ionized water 24 h before the sample manufacturing. A constant water-to-binder mass (W/B) ratio of 0.4 was adopted for preparing the pastes. To investigate the effect of metakaolin, the Na_2_O content and modulus (SiO_2_/Na_2_O molar ratio) in activator were kept constant at 6% of mass of binder and 1.0, respectively. In addition, two activators with 6% Na_2_O at modulus of 1.5 and 8% Na_2_O at modulus of 1.0 were prepared to study the effects of the activator. The activators with a modulus between 1.0 and 1.5 and high Na_2_O content (4–8%) exhibited a good activation efficiency on alkali-activated slag according to a previous study [4]. For the preparation of mortars, a constant W/B ratio of 0.45 and a constant binder-to-sand mass ratio of 0.5 were adopted, respectively. Table 2 shows the mix proportion of samples.

For the preparation of alkali-activated slag paste, the slag and metakaolin if used, were placed in a mixing pot and mixed for 1 min. Then, the activator was added and stirred for 2 min until uniform. For the preparation of the mortar, the extra 2 min was needed when the standard sand was added. Then, the mortar was cast into the steel molds with a dimension of 40 mm × 40 mm × 40 mm and covered with a plastic film for 24 h. Afterwards, they were demolded and placed in a standard curing chamber with a constant temperature of 20 ± 2 °C and relative humidity (RH) higher than 95% until tests.

### 2.3. Testing Procedure

The viscosity of the paste was measured by using NM110 viscometer at the constant shear rate. After mixing, the fresh paste was immediately cast into a bottle with a height of 65 mm and a diameter of 45 mm to perform the test. The rotation speed of 6 rpm was adopted. The test range is between 1.0 and 100 Pa·s, and the data was recorded every 30 s until the paste viscosity exceeded the range of the viscometer.

Every six mortar samples at different curing ages of 3 days, 7 days, 14 days, 28 days, 56 days, and 90 days were used for the compressive strength test. The loading rate was 2.4 kN/s.

**Figure 3:**
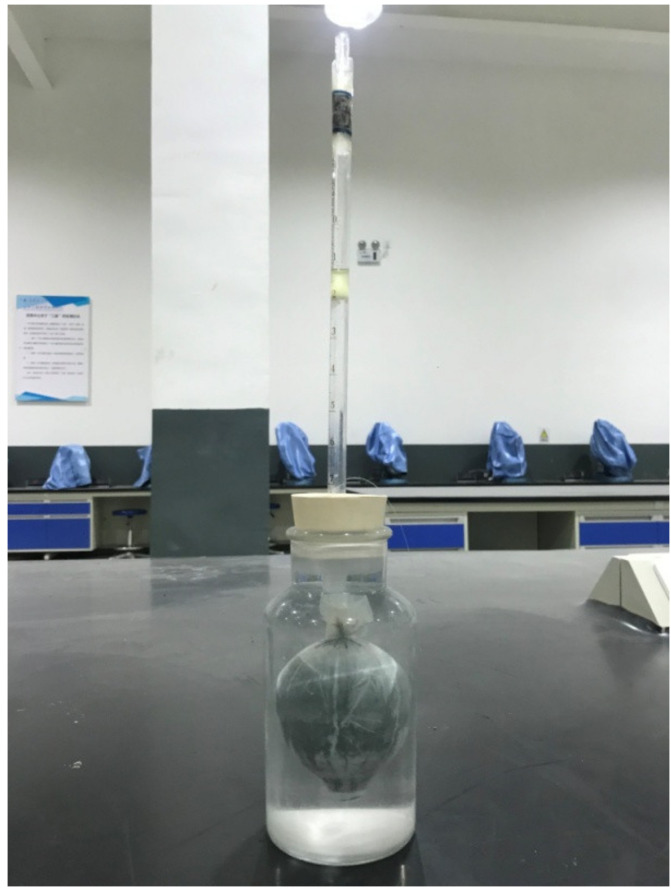
Illustration of testing set-up for the measurements of autogenous shrinkage of alkali-activated slag.

The measurement of the autogenous shrinkage of the mortar was in accordance with the previous studies [28]. As illustrated in Figure 3, the autogenous shrinkage of alkali-activated slag mortar was characterized through the change in the volume of the sample in a water-filled jar, which was reflected by the variation of the liquid level in a glass tube connected with the container. Based on the above principle, about 200 mL of the fresh mortar was cast into a balloon. Then, air bubbles were removed and the balloon was sealed. Afterwards, the balloon was floated to the center of the jar with a thread and water was added into the jar. A rubber lid with a glass tuber adapted from a pipet was used and the neck of the jar was sealed with paraffin to avoid evaporation of the water in the jar. Finally, the water was injected into the glass tube and a drop of oil was added to prevent the evaporation of water in the glass tube. The autogenous shrinkage measurement was conducted in a curing chamber with a constant temperature of 20 ± 2 °C. The variation of the liquid level was recorded every 2 h in the first 24 h and every 6 h in the second 24 h. From the third day, it was recorded daily. The average value of the three specimens for each group was reported.

The drying shrinkage measurement of alkali-activated slag mortar was conducted in accordance with JC/T603-2004 [29]. The prismatic samples with a dimension of 25 mm × 25 mm × 280 mm were adopted. After demolding, the specimens were cured at the curing chamber with a constant temperature of 20 ± 2 °C and a RH higher than 95% for another 3 d. Then, the initial length was measured using a comparator and the specimens were sent to another curing chamber with a constant temperature of 20 ± 2 °C and a RH of 50 ± 4% for the follow-up measurement. The average value of the three specimens for each group was reported.

XRD test was conducted on powdered samples. The acquired slag and metakaolin were separated using a sieve with a diameter of 45 μm. XRD analysis was conducted by using a X-ray diffractometer (D /Max-5A, Rigaku USA, Danvers, MA, USA) between 10 and 70° 2θ at a scan speed of 3.0°/min. Fourier transform infrared spectroscopy (FTIR) and nitrogen adsorption/desorption tests on the paste samples of alkali-activated slag with metakaolin were carried out. The samples that were cured for 28 days were broken. Then, the absolute ethyl alcohol was used to stop the reaction of crushed samples. Afterwards, the powdered samples were placed in the vacuum drying chamber at a constant temperature of 60 °C for 72 h. The crushed sample with a diameter of about 0.8 mm was used for the nitrogen adsorption test. Moreover, the powdered samples were used for FTIR test by using a Thermo-Scientific IS10 FTIR (Waltham, MA, USA) work-station and the disc method with a powdered sample-to-KBr mass ratio of 1:200. The resolution of 2 cm^−1^ with 32 scans was adopted in this study. ASAP 2020 PLUS (MICROMERITICS Instrument Ltd., Atlanta, GA, USA) was used to determine the specific surface area and pore structure of the sample.

## 3. Results and Discussion

### 3.1. Viscosity and Initial Setting Time of Alkali-Activated Slag Pastes

**Figure 4:**
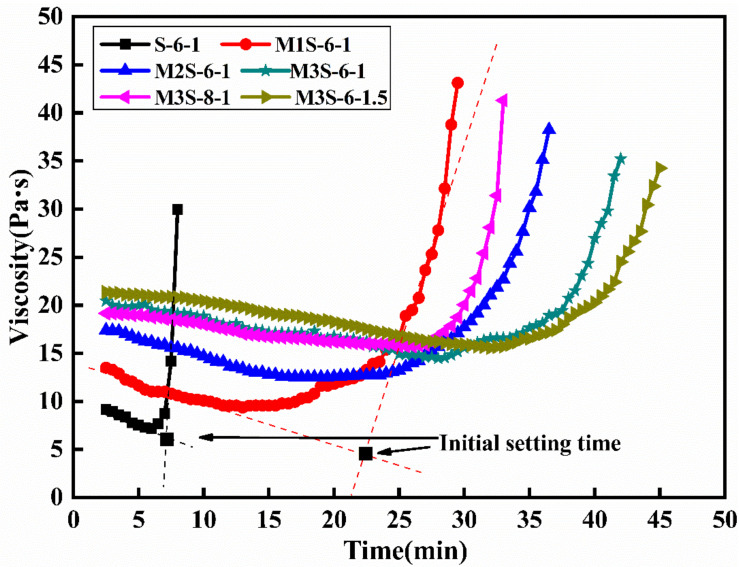
Viscosity of alkali-activated slag with different metakaolin content, activator modulus, and Na_2_O contents.

Figure 4 depicts the influences of metakaolin, activator modulus, and Na_2_O content on the viscosity of alkali-activated slag. It can be found that the viscosity of all samples decreases first, then rapidly increases with time. In addition, the incorporation of metakaolin results in the increase in viscosity of the pastes at an early stage. Compared with slag, metakaolin is finer and has a higher water absorption capacity, which leads to the increase in the viscosity of the paste [30]. It can be seen from Figure 4 that increasing Na_2_O content leads to the slight decrease in the viscosity of alkali-activated slag with 30% metakaolin. The activator with a higher Na_2_O content would introduce more dissolved OH^−^ into the activator, which would attack the Si–O–Si and Si–O–Al bonds in metakaolin and lead to its depolymerization [25]. Therefore, higher Na_2_O content would promote the rapid dissolution of metakaolin and the formation of aluminosilicate gels. Consequently, the water absorption of metakaolin particles is mitigated. Furthermore, the increase in activator modulus results in the increase in viscosity. When the activator modulus increases from 1 to 1.5, the initial viscosity of alkali-activated slag with 30% metakaolin increases by about 4.4%, which could be attributed to more silicates added into the system that result in the increase in viscosity [31].

**Figure 5:**
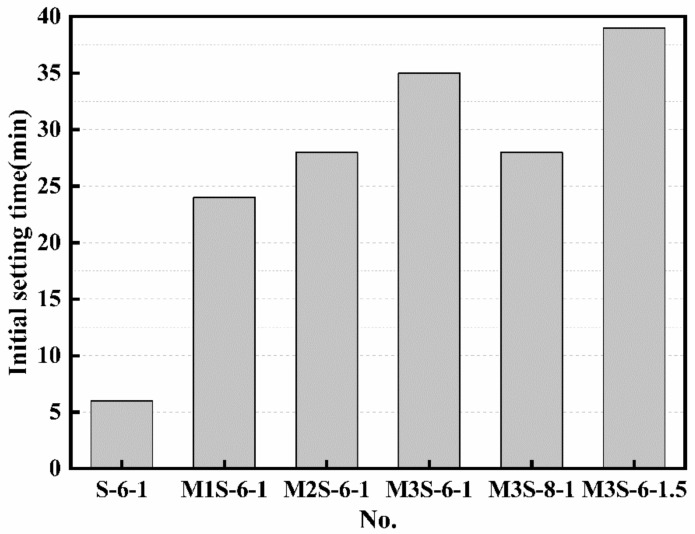
Initial setting time of alkali-activated slag with different metakaolin contents, activator modulus, and Na_2_O contents.

Previous studies considered that this sharp increase in the viscosity of the paste corresponded to the initiation of plastic loss of the paste, which could be regarded as a symbol of the initial setting of the paste [32,33]. Therefore, as illustrated in Figure 4, the initial setting time of alkali-activated slag was regarded as the time corresponding to the intersection point of the two tangent lines of the viscosity curves. Figure 5 depicts the initial setting time of alkali-activated slag blended with metakaolin with different activator modulus and concentrations. Consistent with the previous research [6,25], the increase in metakaolin dosage from 0% to 30% leads to the increase in the initial setting times of the pastes, from 6 min to 35 min. The replacement of slag with metakaolin results in the decrease in CaO from slag and the increase in Al_2_O_3_ and SiO_2_ dosage from metakaolin. On the other hand, the increase in activator concentration contributes to the shortening of the initial setting time of alkali-activated slag with 30% metakaolin. The initial setting time of the sample with 8% Na_2_O content decreased by 22.8%, compared with that of the sample with 6% Na_2_O content. In addition, the initial setting time of alkali-activated slag with 30% metakaolin is prolonged with the increase in the activator modulus, which is consistent with the previous research [5]. Li et al. [25] considered that the lower bonder energy of Ca–O than those of Si–O and Al–O bonds play a dominant role in the hardening of alkali-activated materials, and a higher concentration of the activator is needed to promote the dissolution of metakaolin particles by breaking down the Si–O and Al–O bonds. In the alkaline solution, the increase in the activator modulus results in the relative decrease in OH^−^ ion, thus inducing the increase in high-polymeric silicate with lower activation energy [5]. As a result, the increase in the modulus of the activator from 1.0 to 1.5 leads to the prolonging of the initial setting of the sample in this study.

### 3.2. Compressive Strength Development of Alkali-Activated Slag Mortars

**Figure 6:**
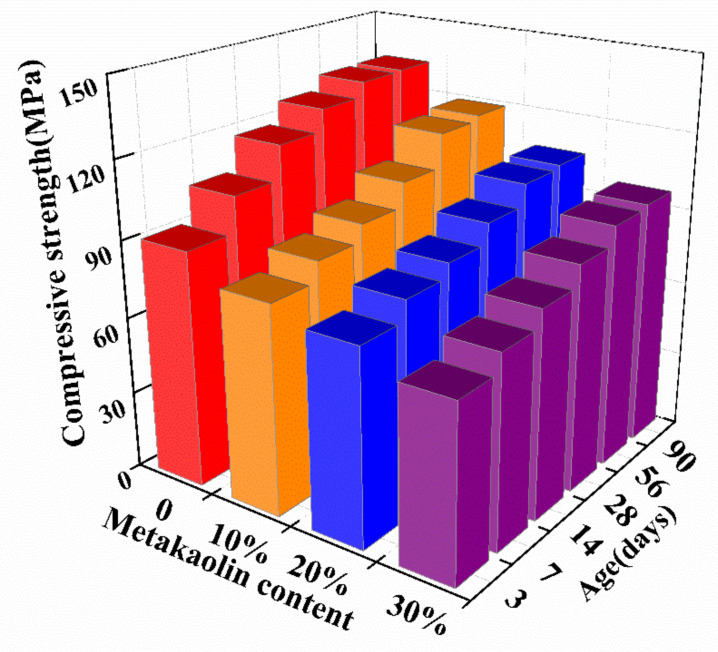
Effect of metakaolin content on the compressive strength of alkali-activated slag mortar.

Figure 6 shows the effect of metakaolin dosage on the compressive strength development of alkali-activated slag mortar with constant activator concentration and modulus. It can be found that the compressive strength of the specimen decreases as the metakaolin dosage increases. For instance, the compressive strengths of the samples cured for 3 days with 10%, 20%, and 30% metakaolin are 11.3%, 17.3%, and 26.4%, respectively, lower than that of the sample without metakaolin; the compressive strengths of the 90 days cured samples with 10%, 20%, and 30% metakaolin are 10.2%, 20.6%, and 27.2%, respectively, lower than that of the sample without metakaolin. This is attributed to the decrease in CaO content in the binders induced by replacement of slag with metakaolin [25]. On the other hand, Huseien et al. [30] reported that alkali-activated slag–metakaolin binary system exhibited a lower early mechanical strength development than the pure alkali-activated slag one, but obtained a comparable mechanical performance with increasing curing age. This could be attributed to the promotion of the formation of N-A-S-H gel induced by the increases in Al_2_O_3_ and SiO_2_ contents from metakaolin. However, in this study, a different trend in the compressive strength development from the previous research by Huseien et al. [30], could be attributed to a lower curing temperature adopted in the current study, which, to some extent, impacts the reaction process of geopolymerization of metakaolin. Based on the strength results from this study, it can be concluded that the incorporation of metakaolin results in the decrease in the compressive strength. However, for an alkali-activated slag system with high mechanical performance, to strike a balance among controlling setting times, mitigating shrinkage, and mechanical property is more important for the future application of performance-based alkali-activated materials. Therefore, as shown in Figure 6, the 28 days compressive strength of the sample with high metakaolin content (30%) and W/B ratio (0.45) exceeds 60 MPa, which indicates the promising potential of using this type of material in application.

**Figure 7:**
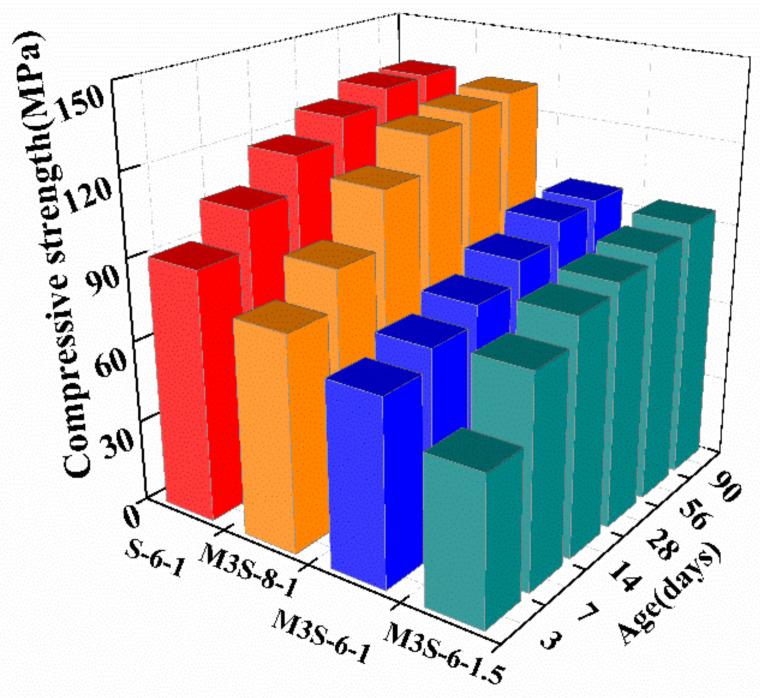
Effects of activator concentration and activator on the compressive strength of alkali-activated slag mortar with 30% metakaolin.

Figure 7 illustrates the influences of activator concentration and modulus of alkali-activated slag mortar containing 30% metakaolin. It can be found that the increase in activator concentration results in an increase in the compressive strength of alkali-activated slag mortar. In addition, the activator concentration has a significant influence on the improvement of the compressive strength at the early age. The compressive strengths of the 3 days and 14 days cured samples with the activator of a Na_2_O content of 8%, increased by 16.4% and 40%, respectively, compared with that of the sample added the activator with a Na_2_O content of 6%. On the other hand, from Figure 7, the increase in activator modulus from 1.0 to 1.5 leads to the reduction in the compressive strength of alkali-activated slag mortar, which is consistent with the results in the previous studies [34,35]. Using the activator with high modulus increases the silicate content in the system which induces the higher surface tension of the solution, thus hindering the dissolution of precursor particles [34,36].

### 3.3. Autogenous and Drying Shrinkage of Alkali-Activated Slag Mortar

**Figure 8:**
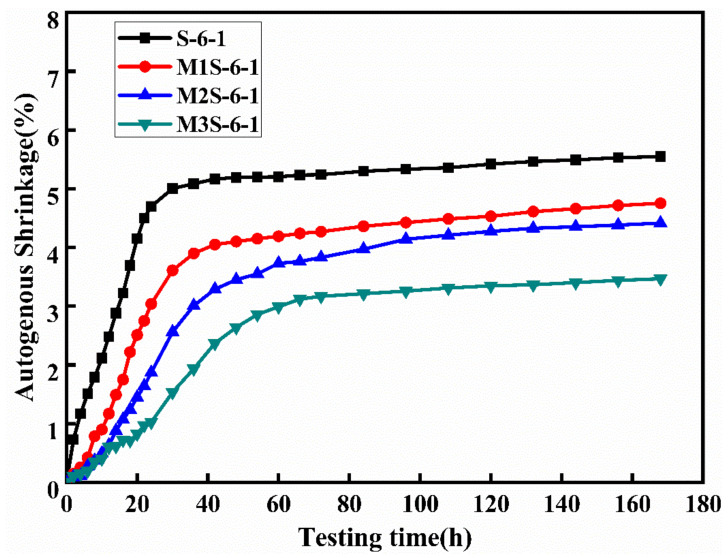
Effects of metakaolin on the autogenous shrinkage of alkali-activated slag mortar.

The autogenous shrinkage behavior of alkali-activated slag is regarded as the combined consequence of the effects of chemical autogenous and self-desiccation shrinkages, which occurs at the early reaction stage of the sample; its development is induced by consuming water during the reaction [20]. Figure 8 shows the influence of metakaolin dosage on the autogenous shrinkage of alkali-activated slag mortar. The samples without metakaolin have a higher autogenous shrinkage among other samples with different metakaolin contents, indicating that the inclusion of metakaolin in alkali-activated slag contributes to mitigating autogenous shrinkage of alkali-activated slag, which is consistent with the results reported by Li et al. [6,17]. In addition, the development of autogenous shrinkage is relatively slow with the incorporation of metakaolin, such as the time to stable stage is prolonged. This corresponds to the results in Section 3.1, that the inclusion of metakaolin retard the reaction.

As discussed previously, incorporating metakaolin could effectively retard the reaction process of alkali-activated slag and influence the formation of reaction products. As the metakaolin dosage increases, more N-A-S-H gel is formed, thereby decreasing the intensity of the chemical shrinkage [6]. The mitigation mechanism of metakaolin on the autogenous shrinkage of alkali-activated slag will be further discussed in the following sections.

**Figure 9:**
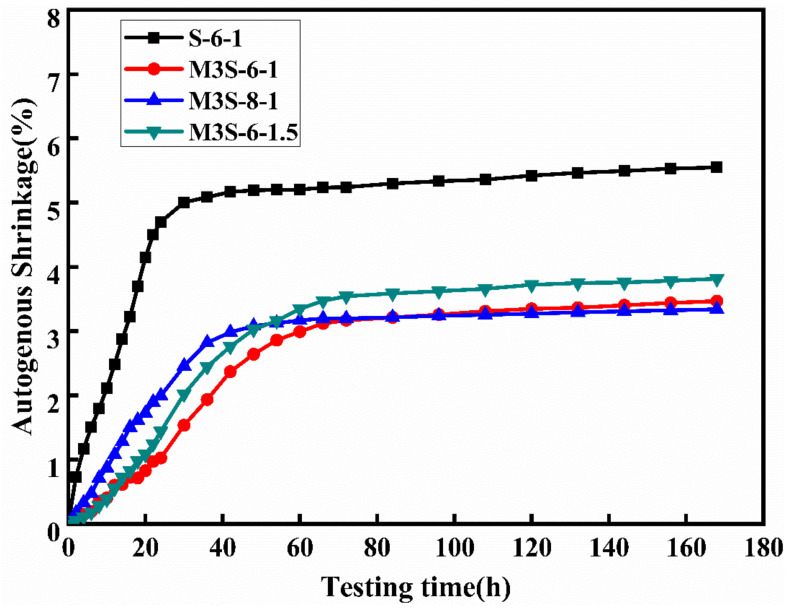
Effects of activator concentration and modulus on the autogenous shrinkage of alkali-activated slag mortar with 30% metakaolin.

Figure 9 depicts the influences of activator concentration and modulus on the autogenous shrinkage of alkali-activated slag mortar with 30% metakaolin. It can be found that the increases in activator concentration and modulus have a significant influence on the early autogenous shrinkage of alkali-activated slag mortar with 30% metakaolin. The samples activated by alkaline solution with higher Na_2_O content (8%) shrink more intensely. This corresponds to the rapid dissolution of precursor particles in a higher alkaline environment. However, after 60 h, the activator concentration shows less influence on the autogenous shrinkage.

**Figure 10:**
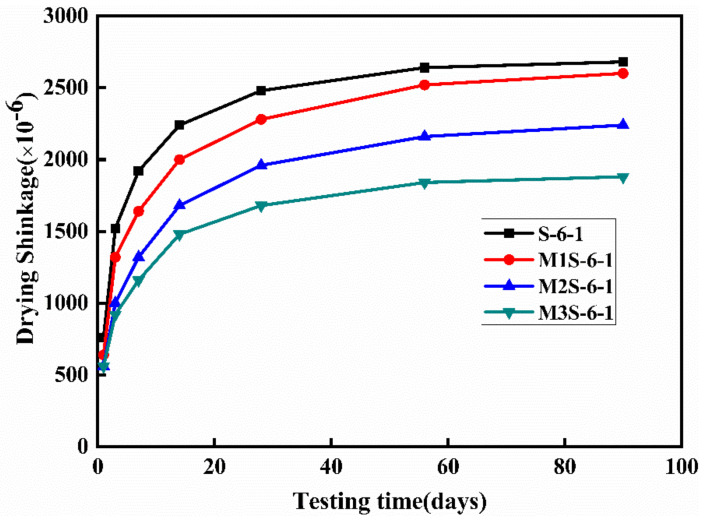
Effect of metakaolin content on the drying shrinkage of alkali-activated slag mortar.

**Figure 11:**
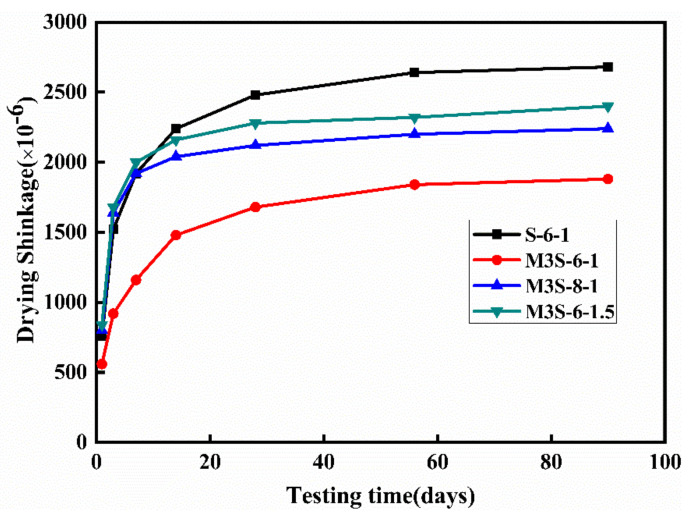
Effects of activator concentration and modulus on the drying shrinkage of alkali-activated slag mortar with 30% metakaolin.

The effect of metakaolin content on the drying shrinkage of alkali-activate slag mortar is depicted in Figure 10. The sample without metakaolin exhibits the highest drying shrinkage rate at the early age and shrinkage magnitude. Additionally, it can be found that after 28 days curing, the drying shrinkage curves of all samples trend to be stable and the drying shrinkage decreases with the increase in metakaolin content. The drying shrinkage at 90 days of the sample with 30% metakaolin is about 29.9% lower than that of the sample without metakaolin. On the other hand, Figure 11 shows the influences of activator concentration and modulus on the time-dependent drying shrinkage of alkali-activated slag mortar containing 30% metakaolin. The increases in activator concentration and modulus result in the increases in the drying shrinkage rate and shrinkage magnitude of the samples. Furthermore, it can be observed that after 28 days curing, the increasing rate of the drying shrinkage of all samples becomes stable. These observations are consistent with the previous research [22,37,38]. Furthermore, it is noted that the time-dependent shrinkage behavior is related with water evaporation [18,21], the nature of reaction products [6], and the pore structure [39]. Therefore, the results of the time-dependent autogenous and drying shrinkage of alkali-activated slag will be discussed with the microstructure analysis.

### 3.4. FTIR Analysis

**Figure 12:**
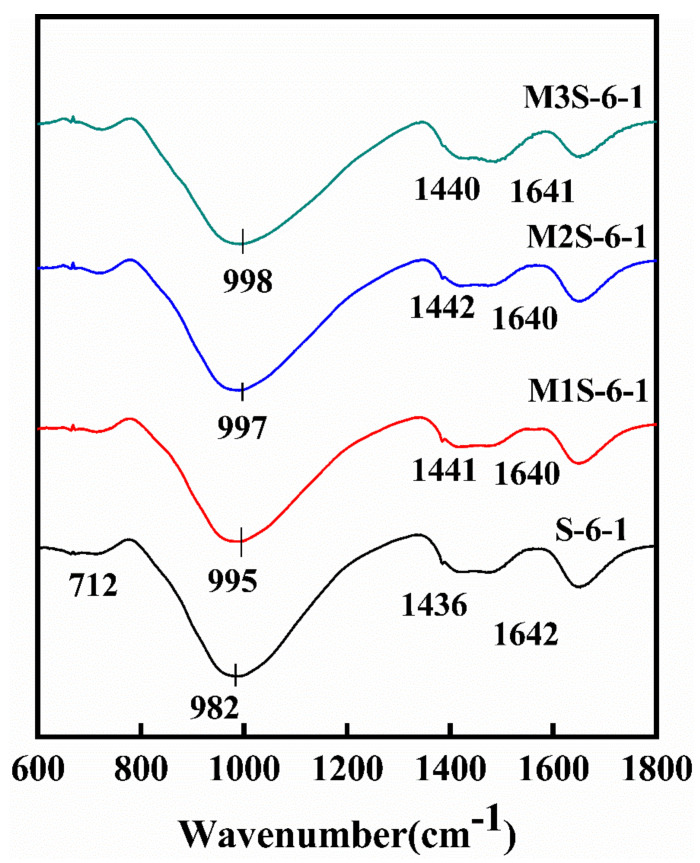
FTIR spectra of alkali-activated slag with different metakaolin contents.

To elaborate the influence of metakaolin on the phase change in alkali-activated slag samples, FTIR analysis was performed with a focus on bands of Si–O–T (T = Si, Al), H–O–H, and C–O, which corresponds to the reaction product gels, chemically bonded water, and CaCO_3_, respectively. Figure 12 shows the influence of metakaolin on the phase change in the samples in the range of 400–2000 cm^−1^. In all samples, there is a major absorption peak at about 1000 cm^−1^, corresponding to the non-uniform stretching vibration of Si–O–T (T = Si, Al) bond due to the dissolution of aluminosilicate phases in precursors. As the metakaolin content increases from 0 to 30%, the wavenumber of this band shifts to a higher wavenumber from 982 cm^−1^ to 1001 cm^−1^. This is due to more Al phase participating in the reaction, which is from metakaolin, thus resulting in the formation of C-A-S-H gel with high polymerization degree [25,40]. On the other hand, a decreasing trend of the intensity of this band with the increase in the metakaolin dosage is observed as well. This implies the decrease in the reaction product amount, which can also explain the findings that the compressive strength decreases with the increase in the metakaolin content, as mentioned in Section 3.2. Furthermore, it can be found that the intensity of the band at about 710 cm^−1^, attributed to the bending vibration of Al–O–Si bond, increases with the increase in metakaolin content. This could be related to the incorporation of more metakaolin [41,42].

**Figure 13:**
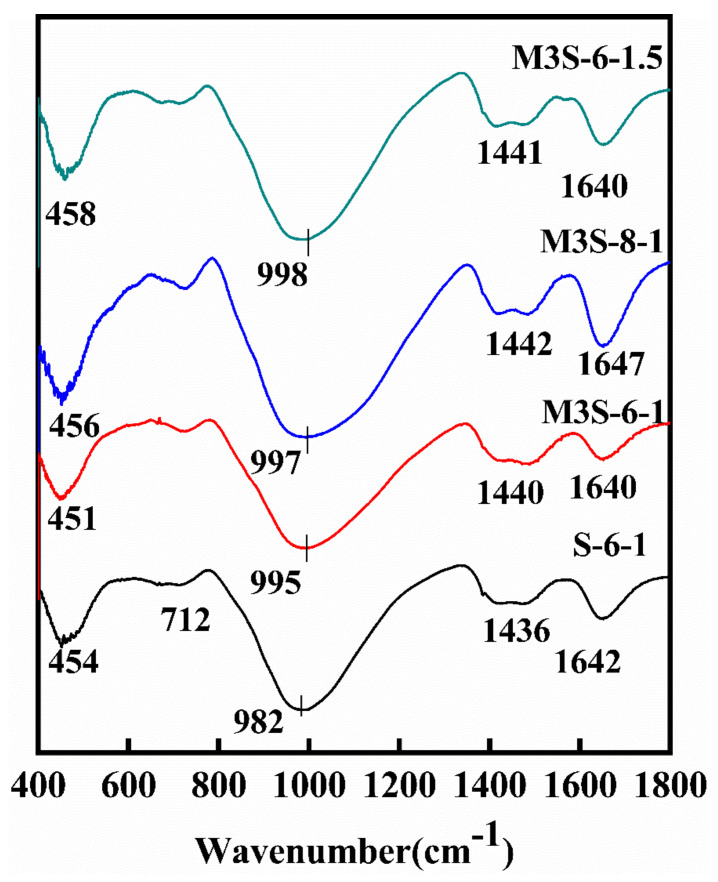
FTIR spectra of alkali-activated slag containing 30% metakaolin with different activator concentrations and modulus.

Figure 13 illustrates the influences of activator concentration and modulus on the phase change in alkali-activated slag containing 30% metakaolin. With the increases in the activator concentration and modulus, the asymmetric stretching Si–O–T bond shift to a higher wavenumber. This is because more silicate is introduced to the system, thus resulting in the formation of Si-rich gel phase [42]. Moreover, the band at about 1000 cm^−1^ becomes more intensive and wider with increasing activator concentration, indicating a higher degree of the reaction. It is noted that the sample with the activator with a Na_2_O content of 8% has a sharp bond at about 710 cm^−1^, which is attributed to the band vibration Al–O–Si bond, indicating that more metakaolin participates in the reaction due to more dissolution of metakaolin at a higher alkaline environment. Furthermore, compared with the influence of activator concentration, increasing the activator modulus could directly introduce more silicate into the system, which results in the decrease in Ca/Si and Al/Si ratios in gel, thus promoting a high level of the formation of Si-rich gel phase, which results in the occurrence of the widening of the shoulder at about 1000 cm^−1^ and the increase in the intensity of the asymmetric stretching vibration of the bond at about 450 cm^−1^, when the activator modulus increases.

### 3.5. Isothermal Adsorption/Desorption

**Figure 14:**
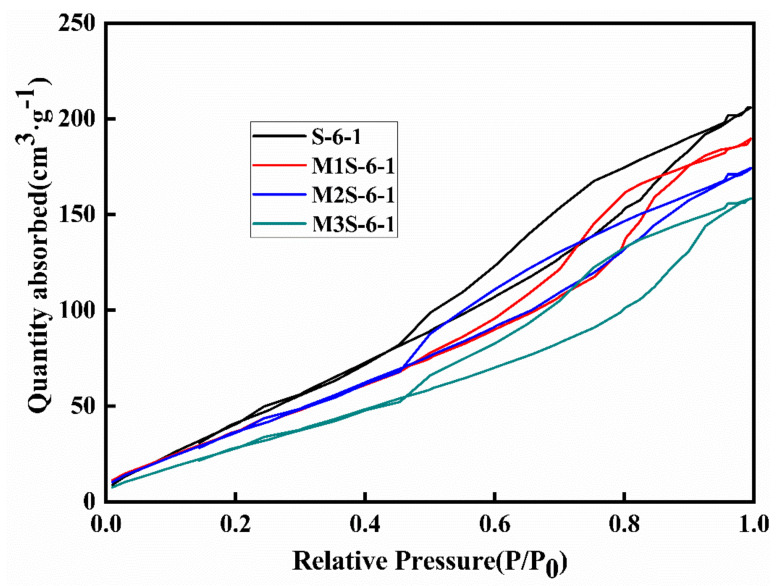
Isothermal adsorption–desorption curves of alkali-activated slag with different metakaolin contents.

**Figure 15:**
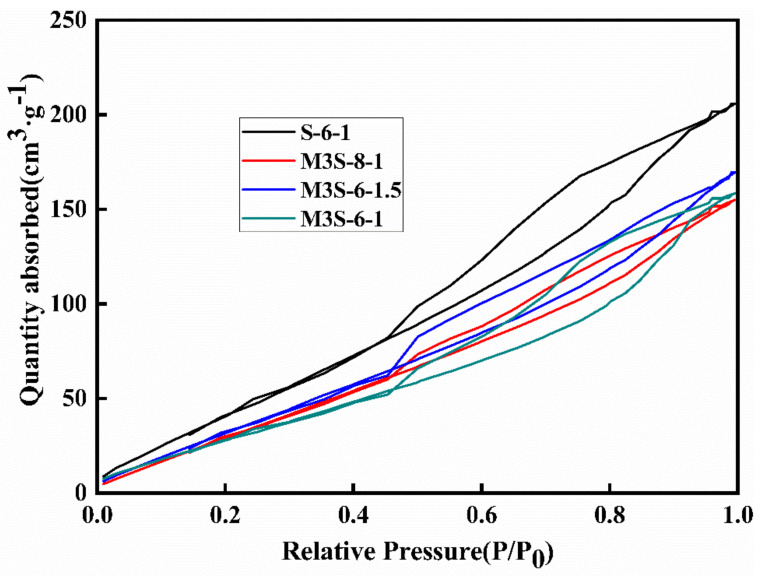
Isothermal adsorption–desorption curves of alkali-activated slag containing 30% metakaolin with different activator concentrations and modulus.

Figure 14 and Figure 15 show the isothermal adsorption–desorption curves of the samples. It can be found that all isothermal adsorption–desorption curves can be classified into the Type IV isothermals based on the previous research [21,43], which indicates that it is a mesoporous structure. It can be found that when the relative pressure (RP) is less than 0.5, the adsorption and desorption curves of all samples are overlapped and reversible. However, the isotherms present a significant hysteresis loop when the RP exceeds 0.5, which is due to the N_2_ adsorption occuring at the annular membrane level on the middle pore wall, while N_2_ desorption is at the spherical meniscus level at the middle pore mouth. Thus, this hysteresis loop can be used to evaluate the pore characteristic of the samples [21,44]. From Figure 14, it can be found that metakaolin has less influence on the slope of the isotherm curves and exhibits a negative effect on the adsorption capacity of adsorbents, implying that the inclusion of metakaolin influences the pore structure of the sample. With the increase in the metakaolin dosage from 0 to 30%, the adsorption quantity of the sample decreases, regardless of adsorption or desorption branch. This could be attributed to the presence of coarser pore structure induced by incorporations of metakaolin. Besides, the area of the hysteresis loop increases with increasing metakaolin content, which is related to the formation of narrow necks in the pore networks.

Similarly, the variation of activator concentration and modulus also influence the isotherms of the samples. However, the activator concentration has less influence on the adsorption quantity and the area of the hysteresis loop tends to be smaller when the activator concentration increases. This indicates the positive influence of increasing Na_2_O content for refining the pore structure of the sample. On the other hand, the increase in the activator modulus results in the increase in the isothermal adsorption–desorption quantity of adsorbents and area of the hysteresis loop, demonstrating the coarsening of the pore structure of the sample with higher activator modulus.

**Figure 16:**
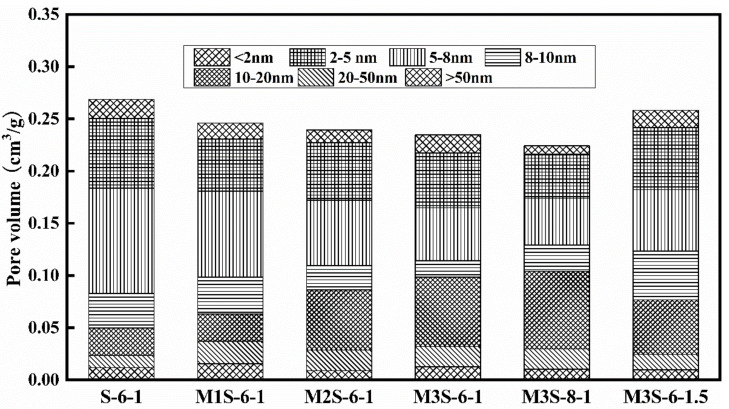
Pore size distribution of alkali-activated slag.

To further elaborate the influences of metakaolin and activator concentration and modulus on the pore structure of alkali-activated slag, the micro and meso-pore size distributions are shown in Figure 16. The Barret-Joyner-and-Halenda (BJH) method was adopted in this study to determine the pore size distribution in the meso-pore range (2–50 nm) [45]. It can be seen from Figure 16 that in the sample without metakaolin, the volume of the pore less than 10 nm is 2.204 cm^3^/g, accounting for 81.5% of the total pore volume. When 10%, 20%, and 30% metakaolin are introduced, their pores less than 10 nm are 1.858, 2.354, and 2.301cm^3^/g, respectively, accounting for 74.3%, 64.0%, and 58.0%, respectively. More specifically, the volume of the pore in a range of 5–8 nm decreases as well with increasing metakaolin content. It is necessary to point out that based on the previous research [46,47], there has been a classification of different pores: gel pore (2–50 nm), capillary pore (10–100 nm), and macro pore (>1000 nm), namely. However, in this study, it is difficult to define the type of the pores (gel pores or capillary pores) with a size between 10 to 20 nm, although the pores with a size between 5 to 10 nm could be defined as gel pores [39]. As shown in Figure 16, the increase in the volume of the pores with a size range between 5 to 8 nm and the increase in the volume of the pores in a size range between 10 to 20 nm are a result of the increase in metakaolin dosage. It is believed that the more capillary pores, the greater the drying shrinkage [48]. However, as reported previously in this study, the increase in metakaolin contributes to mitigating drying shrinkage of the sample. Therefore, in this study, it is considered that the 10–20 nm pore could be defined as a gel pore, rather than a capillary pore. In summary, in combination with the results of the FTIR analysis, the increase in metakaolin dosage in the binders results in the formation of reaction products with a high Si amount, thus leading to the coarsening of the pore structure [49].

In addition, it can be found that the increase in Na_2_O content reduces the total pore volume, which is related with the improvement of the reaction degree with a higher alkaline condition. Higher alkaline solution, as discussed previously, could promote the dissolution and breaking down of metakaolin particles, thus inducing the coarsening of the resultant gel pores. Moreover, the increase in the activator modulus from 1.0 to 1.5 results in the increase in the total pore volume, but has less effect on the variation of the pore size distribution. This could be attributed to more soluble silica being introduced in the system with increasing activator modulus, which increases the viscosity of the mixture, thus inducing the porosity [50].

### 3.6. Relationship between Drying Shrinkage and Pore Structure Characteristic of Alkali-Activated Slag

Generally, the time-dependent drying shrinkage behavior is strongly dependent upon the nature of the pore structure. Dry shrinkage could be attributed to the loss of water inside the cement matrix, which involves with capillary tension force [51], liquid separation pressure, surface energy, and loss of interlayer water [21]. Thus, in this section, the relationships between drying shrinkage and pore structure characteristic of alkali-activated slag with different metakaolin contents, and activator concentrations and modulus are discussed.

**Figure 17:**
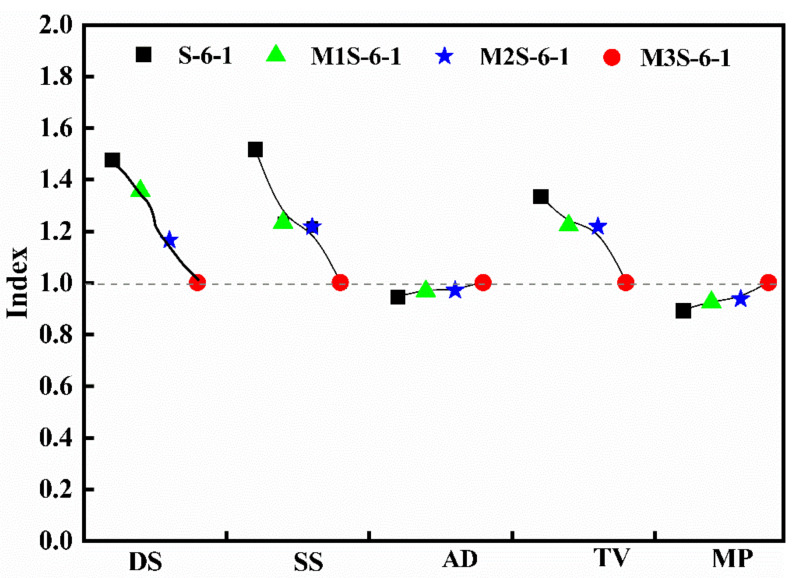
Influence of metakaolin on the drying shrinkage and pore structure characteristic parameters of alkali-activated slag.

**Figure 18:**
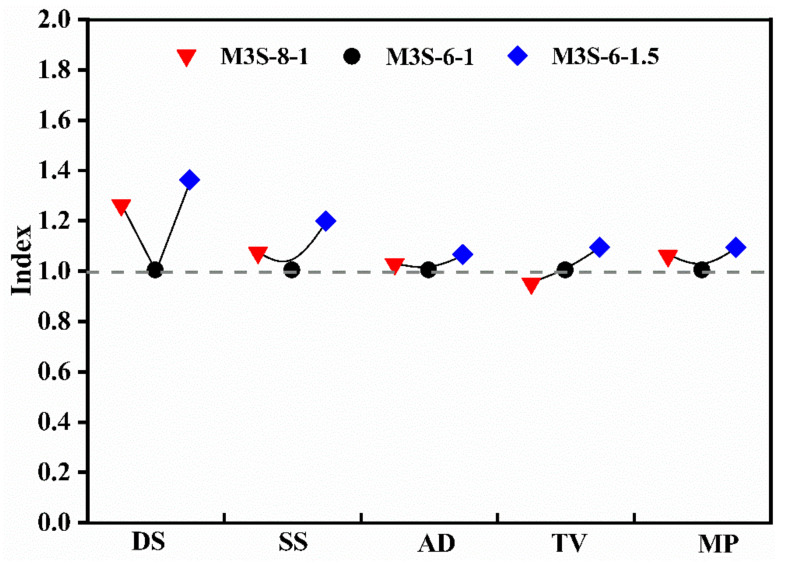
Influences of activator concentration and modulus on the drying shrinkage and pore structure characteristic parameters of alkali-activated slag containing 30% metakaolin.

Figure 17 and Figure 18 illustrate the influences of metakaolin contents, activator concentration, and modulus on the drying shrinkage (DS) and the pore structure parameters, including specific surface area (SS), average diameter (AD), total volume (TV), and mean pore size (MP) of alkali-activated slag sample cured for 28 days. The indexes of the DS, SS, AD, TV, and MP are defined as the ratios of the corresponding values of other samples to those of the sample M3S-6-1. From Figure 17, there are decreasing trends of drying shrinkage, pore specific surface area, and total pore volume with the increase in metakaolin content, while the pore average diameter and mean pore size increase when the metakaolin dosage rises. This clearly indicates that the incorporation of metakaolin reduces the porosity, while coarsens the pore structure, which mitigates the dry shrinkage. This is consistent with the above-mentioned results and discussion.

Furthermore, from Figure 18, it can be found that the increase in the activator concentration results in increases in the drying shrinkage, specific surface area, average diameter, and mean pore size, but a decrease in the total pore volume. This also demonstrates that increasing the activator concentration has a similar effect to the inclusion of metakaolin. The increase in activator modulus results in the increases in drying shrinkage and pore structure characteristic parameters. Although increasing activator modulus coarsens the pore structure of the sample containing 30% metakaolin, there is still a higher drying shrinkage observed. This indicates that the influence mechanism of increasing activator modulus is different from that of metakaolin. Ye et al. [37] concluded that the activator modulus could influence the elastic modulus of pure alkali-activated slag, thereby impacting the drying shrinkage. Thus, in this study, it could be considered that the variation of the nano-mechanical property induced by changing the activator modulus has a dominant influence on the drying shrinkage of alkali-activated slag containing 30%.

## 4. Conclusions

This study investigated the influence of metakaolin on the shrinkage behaviors of alkali-activated slag, and further evaluated the effects of activator parameters on the mitigation mechanism of alkali-activated slag containing metakaolin. Based the presented results and discussion, the main conclusions can be drawn:

1. The inclusion of metakaolin improved the initial viscosity and setting time of alkali-activated slag. However, increasing activator concentration resulted in the reduction in the initial viscosity and setting time of alkali-activated slag containing 30% metakaolin, and the initial viscosity and setting time are both increased with the increase in activator modulus from 1 to 1.5;
2. The increase in metakaolin content led to the decrease in the compressive strength development of alkali-activated slag mortar. Increasing the activator concentration contributed to the improvement of the compressive strength of alkali-activated slag containing metakaolin. However, activator modulus had a less significant influence on the compressive strength;
3. The autogenous and drying shrinkages of alkali-activated slag mortar were both mitigated by the inclusion of metakaolin. The increase in activator dosages and modulus both resulted in the increase in the magnitudes of the autogenous and drying shrinkage of alkali-activated slag containing 30%;
4. The inclusion of metakaolin induced the decrease in reaction products and promote the formation of the reaction product with high polymerization degree. Higher activator dosage and modulus contributed to the dissolution and decomposition of metakaolin, thus facilitating the formation of the reaction products;
5. The inclusion of metakaolin into alkali-activated slag and increasing the activator dosage decreased the total pore volume, but induced the coarsening of the pore structure. There was a different mechanism behind the influence of activator modulus on mitigating the time-dependent behavior of the alkali-activated slag-metakaolin binary system.

## Figures and Tables

**Table 1:** Chemical composition of slag and metakaolin.

	K_2_O	Na_2_O	SO_3_	SiO_2_	Fe_2_O_3_	Al_2_O_3_	CaO	MgO	TiO_2_	LOI
Slag	0.83	0.73	0.13	35.88	0.46	10.65	33.54	11.43	1.14	1.3
Metakaolin	0.44	0.41	-	49.78	0.93	34.63	-	2.58	1.01	1.1

**Table 2:** Mix proportions.

**Paste Sample**						
**No.**	**Slag (wt.%)**	**Metakaolin (wt.%)**		**Modulus of Activator**	**Na_2_O (%)**	**W/B Ratio**
S-6-1	100	0		1	6	0.4
M1S-6-1	90	10				
M2S-6-1	80	20				
M3S-6-1	70	30				
M3S-8-1	70	30		1	8	
M3S-6-1.5	70	30		1.5	6	
**Mortar Sample**						
**No.**	**Slag (wt.%)**	**Metakaolin (wt.%)**	**Fine Aggregate (wt.%)**	**Modulus of Activator**	**Na_2_O (%)**	**W/B Ratio**
S-6-1	33.33	0	66.67	1	6	0.45
M1S-6-1	29.667	3.333	66.67			
M2S-6-1	26.664	6.666	66.67			
M3S-6-1	23.331	9.999	66.67			
M3S-8-1	23.331	9.999	66.67	1	8	
M3S-6-1.5	23.331	9.999	66.67	1.5	6	

## Data Availability

The data presented in this study are available on request from the corresponding author.
